# Predicting Adverse Neurodevelopmental Outcomes in Premature Neonates with Intrauterine Growth Restriction Using a Three-Layered Neural Network

**DOI:** 10.3390/diagnostics15010111

**Published:** 2025-01-05

**Authors:** Anca Bivoleanu, Liliana Gheorghe, Bogdan Doroftei, Ioana-Sadiye Scripcariu, Ingrid-Andrada Vasilache, Valeriu Harabor, Ana-Maria Adam, Gigi Adam, Iulian Valentin Munteanu, Carolina Susanu, Iustina Solomon-Condriuc, Anamaria Harabor

**Affiliations:** 1 Head of Neonatal Intensive Care Unit, “Cuza voda” Maternity Hospital, 700038 Iasi, Romania; a.bivoleanu@yahoo.com; 2 Surgical Department, Faculty of Medicine, University of Medicine and Pharmacy “Grigore T. Popa”, 700115 Iasi, Romania; 3 Department of Mother and Child Care “Grigore T. Popa”, University of Medicine and Pharmacy, 700115 Iasi, Romaniaiustina_condriuc@yahoo.com (I.S.-C.); 4 Clinical and Surgical Department, Faculty of Medicine and Pharmacy, ‘Dunarea de Jos’ University, 800216 Galati, Romaniavalentin.munteanu@ugal.ro (I.V.M.);; 5 Department of Pharmaceutical Sciences, Faculty of Medicine and Pharmacy, ‘Dunarea de Jos’ University, 800216 Galati, Romania; adam.gigi20@gmail.com

**Keywords:** artificial neural network, IUGR, preterm, Bailey-3 scale, neurodevelopmental delay

## Abstract

**Background/Objectives**: There is a constant need to improve the prediction of adverse neurodevelopmental outcomes in growth-restricted neonates who were born prematurely. The aim of this retrospective study was to evaluate the predictive performance of a three-layered neural network for the prediction of adverse neurodevelopmental outcomes determined at two years of age by the Bayley Scales of Infant and Toddler Development, 3rd edition (Bayley-III) scale in prematurely born infants by affected by intrauterine growth restriction (IUGR). **Methods**: This observational retrospective study included premature newborns with or without IUGR admitted to a tertiary neonatal intensive care unit from Romania, between January 2018 and December 2022. The patients underwent assessment with the Amiel-Tison scale at discharge, and with the Bailey-3 scale at 3, 6, 12, 18, and 24 months of corrected age. Clinical and paraclinical data were used to construct a three-layered artificial neural network, and its predictive performance was assessed. **Results**: Our results indicated that this type of neural network exhibited moderate predictive performance in predicting mild forms of cognitive, motor, and language delays. However, the accuracy of predicting moderate and severe neurodevelopmental outcomes varied between moderate and low. **Conclusions**: Artificial neural networks can be useful tools for the prediction of several neurodevelopmental outcomes, and their predictive performance can be improved by including a large number of clinical and paraclinical parameters.

## 1. Introduction

Intrauterine growth restriction is a condition where the fetus fails to reach its growth potential in utero, and it is associated with an increased risk of adverse perinatal outcomes and long-term complications such as asphyxia, metabolic disruptions, physical retardation, neurodevelopmental disorders, obesity, hypertension, coronary heart disease, type 2 diabetes, and nephropathy [1,2,3,4]. Clinical observation, imaging, and developmental screening can often predict severe neurodevelopmental outcomes in neonates.

The proposed mechanisms behind the neurological injury in growth-restricted fetuses include neuronal apoptosis, neural inflammation, oxidative stress, excitatory toxicity, disruption of the blood–brain barrier, and epigenetic changes [5].

Prediction of IUGR and adverse neurological outcomes occurrence in fetuses has gained a lot of attention in recent years. A multicenter prospective study conducted by the PORTO group evaluated the risk of adverse early childhood developmental outcomes in children affected by growth restriction during pregnancy, as well as the role of cerebroplacental ratio (CPR) in the prediction of these adverse neurodevelopmental outcomes [6]. Their results indicated that at the age of 3, children who had a subunitary CPR had consistently shorter stature and lower weight, exhibited inferior neurodevelopmental outcomes, as well as significantly worse neurological outcomes in all assessed categories using the Bayley Scales of Infant and Toddler Development, 3rd edition (Bayley-III) [6,7].

The Bayley-III is designed for use with children from 1 month to 42 months of age, allowing for the assessment of early development during infancy and toddlerhood [7]. It underwent validation in various cultural backgrounds and proved overall good reliability [8,9,10].

A recent observational cohort study by Gardella et al. investigated the association between histopathological placental lesions and infant neurodevelopmental outcomes at 2 years of age in a cohort of pregnancies complicated by IUGR [11]. The results from this study indicated that severe maternal vascular malperfusion was associated with an increased risk of neonatal mortality, a high risk of developing major and minor neurodevelopmental sequelae, as well as a high risk for personal-social, hearing, and speech disturbances.

The prediction of adverse neurological outcomes in neonates with IUGR is mainly based on the antenatal screening of this disorder. Thus, maternal risk factors, abnormal Doppler parameters, fetal biometry, and the fetal growth rate are considered parameters with significant impact on the occurrence of adverse perinatal outcomes in such pregnancies, including adverse neurological outcomes [12,13,14].

In recent years, artificial intelligence techniques, including machine learning algorithms and artificial neural networks, have proven effective in predicting various medical disorders, such as small for gestational age fetuses, preeclampsia, HELLP syndrome, and seropositivity for hepatitis viruses [15,16,17]. However, the use of such techniques for the prediction of adverse neurodevelopmental outcomes in infants affected by growth restriction during pregnancy has not been studied before.

Thus, the aim of this retrospective study was to evaluate the predictive performance of a three-layered neural network for the prediction of adverse neurodevelopmental outcomes determined at two years of age by the Bayley-III scale in prematurely born infants affected by IUGR during pregnancy.

## 2. Materials and Methods

This observational retrospective study included premature newborns admitted to the neonatal intensive care unit of a tertiary maternity hospital—‘Cuza-Voda’, Iasi, Romania—between January 2018 and December 2022. The inclusion criteria comprised the following: singleton pregnancies, gestational age between 28 and 37 weeks of gestation, and certain first-trimester pregnancy dating. The exclusion criteria were represented by twin and term pregnancies, incomplete medical records, loss of follow-up, and lack of maternal informed consent.

Prematurely born patients were segregated into two groups depending on the presence of growth restriction during pregnancy: group 1 (with IUGR; n = 27 patients) and group 2 (without IUGR; n = 179 patients).

The patients underwent assessment with the Amiel-Tison scale at discharge, as close as possible to correct 40 weeks of gestation [18]. This scale evaluates the neuro-sensory development, cranial morphology, muscular tone, active and passive muscular movements, spontaneous motor activity, alimentary autonomy, visual fixation, and primary archaic reflexes. This examination allows early detection of newborns with minor, moderate, or severe neurological impairment.

Newborns were further included in a follow-up program that included the newborns’ evaluation using the Bailey-III scale at 3, 6, 12, 18, and 24 months of corrected age [7]. Only cognitive, language, and motor scales were included for analysis in the present study. The Bayley-III raw scores were used to calculate the Cognitive Composite (CC), Language Composite (LC), and Motor Composite (MC) scores. In relation to Bayley scores, mild, moderate, and severe delays were defined as scores below 85 points (more than 1 standard deviation below the mean), below 70 points (more than 2 standard deviations below the mean), and below 55 points (more than 3 standard deviations below the mean), respectively. The Bayley III scale is licensed and requires training.

The following additional data were recorded: demographic and clinical maternal characteristics, gestational age at birth, birth weight, Apgar scores at 1 and 5 min, neonatal complications (retinopathy of prematurity—ROP; intraventricular hemorrhage—IVH; periventricular leukomalacia—PVL; and acute respiratory distress syndrome—ARDS), the need for invasive ventilations, and duration of hospitalization.

We used the Shapiro–Wilk test to check for the normality of the continuous data, and in the case of non-normally distributed variables, we reported the medians and interquartile ranges (IQR) and used the Mann–Whitney U test (Wilcoxon rank-sum test) for comparison. If the continuous variables were normally distributed, we used a t-test for intergroup comparisons and reported the means and standard deviations (SD). The comparisons of categorical variables between groups were performed using Pearson’s χ2 test. *p*-values under 0.05 were statistically significant. STATA SE (17, 2023, StataCorp LLC, College Station, TX, USA) was used for these analyses.

**Figure 1:**
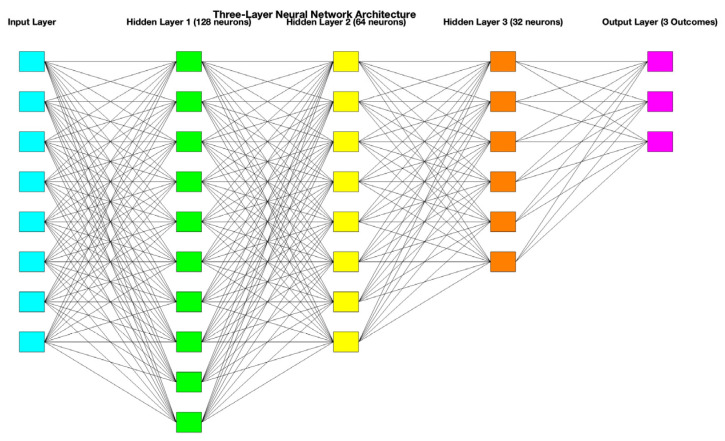
The graphical representation of the three-layered neural network.

All these data were included in a database that was further evaluated using a three-layered neural network developed using Matlab (version R2023a, The MathWorks, Inc., Natick, MA, USA). Standardized data were segregated into 70% training and 30% testing. The artificial neural network comprised one input layer, representing the input features of the dataset (10 neurons corresponding to 10 features), 3 hidden layers (128, 64, and 32 neurons), responsible for extracting hierarchical feature representations, and one output layer (3 neurons) for the prediction of the three neurological outcomes, each with three severity grades. A schematic representation of this three-layered neural network is presented in Figure 1. A grid search approach was employed to optimize the number of neurons in each layer. Also, a 5-fold cross-validation was performed, and the best combination (128, 64, and 32 neurons) was selected based on validation accuracy. A ReLU activation function and an iteration limit of 1000 were employed. Also, a principal component analysis was used to highlight the most informative features.

We calculated the predictive performance of this artificial neural network in relation to the main adverse neurodevelopmental outcomes: severe cognitive, language, and motor delay in IUGR patients. The sensitivity (Se), specificity (Sp), false positive rate (FPR), Matthews correlation coefficient, accuracy, precision, and F1 score were determined.

## 3. Results

In this retrospective study, we analyzed 206 newborns and their mothers. Their clinical characteristics are presented in Table 1. The evaluated groups were relatively homogenous, considering their age (*p =* 0.34), BMI (*p =* 0.76), and level of education (*p =* 0.67). The IUGR group had a significantly higher frequency of maternal smoking during pregnancy (*p*< 0.001), and preeclampsia (*p*< 0.001). The personal history of adverse pregnancy outcomes (preterm birth, preeclampsia, intrauterine growth restriction, emergency cesarean, etc) was also significantly higher for the IUGR group (*p =* 0.01).

On the other hand, the control group, which comprised preterm newborns without IUGR, had higher rates of vaginal infections (22.9 vs. 11.11%), chorioamnionitis (5.02 vs. 3.7%), and prolonged rupture of membranes (5.58 vs. 3.7%), even though we could not determine any significant differences between groups regarding these characteristics (*p* > 0.05).

The neonatal outcomes in the evaluated groups are presented in Table 2. Premature newborns with IUGR had a higher gestational age at birth compared with premature newborns without IUGR, but this difference was not statistically significant (31 (IQR:30–32) vs. 30 (IQR:28–32) weeks of gestation, *p =* 0.06). On the other hand, the birthweight was significantly lower for the first group compared to the second group (1300 (1050–1400) vs. 1400 (1020–1750) grams, *p =* 0.04). No statistically significant differences were determined between these groups regarding Apgar scores at 1 (*p =* 0.92) and 5 min (*p =* 0.95).

When comparing the rates of major neonatal complications, we could not determine any statistically significant differences between groups concerning the rates of ROP (*p =* 0.35), IVH (*p =* 0.21), and PVL (*p =* 0.06).

ARDS occurred in 88.8% of neonates included in the first group, and in 94.97% of neonates included in the second group (*p =* 0.20). In total, 59.25% of IUGR neonates needed invasive mechanical ventilation, while 29.6% of neonates in the control group needed this procedure, and the difference between groups regarding this outcome was statistically significant (*p =* 0.005).

Finally, both groups presented similar hospitalization duration (46.25 ± 20.30 vs. 49.77 ± 29.30 days) without achieving statistical significance (*p =* 0.27).

The neurological and neurodevelopmental outcomes are presented in Table 3. We could not find any statistically significant differences between groups regarding their neurological outcomes at discharge or at 2 years follow-up.

In the final stage of our analysis, we evaluated the predictive performance of a three- layered artificial neural network for the prediction of severe adverse neurodevelopmental outcomes in neonates affected by intrauterine growth restriction, and the results are presented in Table 4.

The three-layered artificial neural network had an overall moderate predictive performance for the prediction of mild forms of cognitive (Sensitivity—Se: 75%, Specificity—Sp: 75%, false positive rate—FPR: 33.3%, and accuracy of 71.4%), motor (Se: 75%, Sp: 90%, FPR: 10%, and accuracy of 83.3%), and language (Se: 62.3%, Sp: 87.5%, FPR: 12.5%, and accuracy of 80%) delays.

On the other hand, the prediction of moderate and severe forms of neurodevelopmental outcomes varied between moderate and low, with the best results being achieved for the prediction of moderate (Se: 66.6%, Sp: 94%, FPR: 5%, and accuracy of 85.7%) and severe (Se: 50%, Sp: 100%, FPR: 0%, and accuracy of 83.3%) cognitive delays.

The model performed best for predicting mild (F1 = 0.75) and moderate cognitive delay (F1 = 0.73), but severe cases of cognitive delay were frequently missed (F1 = 0.66). When used to predict motor delay, the model performed best for the prediction of mild motor delays (F1 = 0.80), while its performance was low for moderate (F1 = 0.50), and severe motor delay (F1 = 0.28). Finally, the model achieved the best performance when used to predict moderate language delays (F1 = 0.66), but had limited capacity in predicting severe language delays (F1 = 0.5).

The proposed artificial neural network had the lowest predictive performance for all grades of language delay in comparison with cognitive and motor delays.

**Figure 2:**
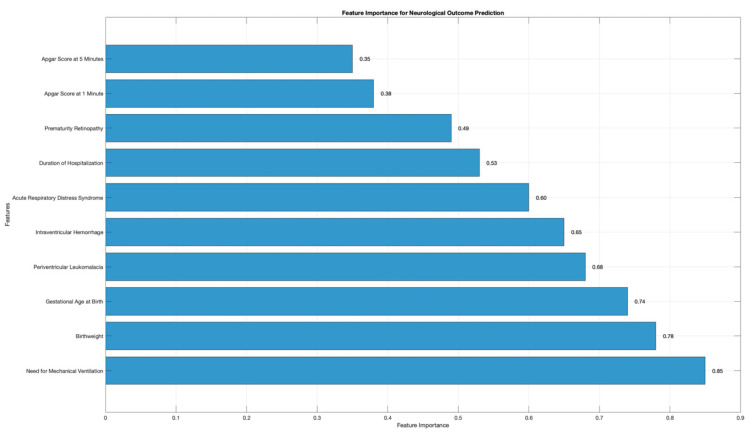
Feature importance for adverse neurological outcomes prediction.

The feature importance for the prediction of the evaluated outcomes is presented in Figure 2. Our results indicated that the need for mechanical ventilation, birthweight, and gestational age at birth were the most important features with an impact on the prediction of adverse neurological outcomes. Additionally, PVL, IVH, and ARDS were predictors with moderate impact. Last but not least, the duration of hospitalization, ROP, and Apgar scores at 1 and 5 min had the least importance in the prediction process.

## 4. Discussion

Intrauterine growth restriction, especially for preterm newborns, can be associated with important adverse neurological outcomes. Recently, the functional taxonomy of preterm birth has been reevaluated, and the need for individualized follow-up of both physical and psychomotor development until two years old of specific premature newborns has been outlined [19]. This study included preterm neonates, with or without intrauterine growth restriction, and followed up on their evolution in the intensive care unit over a 2-year’ timeframe. Our results outlined a higher prevalence of smoking mothers who have a significant personal history of adverse pregnancy outcomes and preeclampsia in the IUGR group compared with the control group.

These risk factors have been previously cited in the literature to have an important influence on the IUGR occurrence in pregnancy. The mechanisms behind the harmful effects of smoking during pregnancy include alteration of immunoregulation, trophoblast function, and placental vasculature development and metabolism [20]. Smoking rates have strong correlations with age and geographic location, but are primarily linked to education. Specifically, women who persist in smoking throughout pregnancy are more prone to having limited educational attainment, low income, and insufficient social support [21]. Our results indicated very high prevalence rates of smoking during pregnancy in the IUGR group (40.7%). Our results regarding the smoking rates were higher than those of other European countries [22]. Although the level of education did not significantly differ between groups, we outline a low rate of high educational level, with a bachelor’s degree being obtained only by 22.22% of mothers who gave birth to growth-restricted neonates. These findings outline the need to promote and implement smoking cessation campaigns among pregnant women, especially in our region.

Both personal history of adverse pregnancy outcomes and preeclampsia have been identified as risk factors for IUGR in many observational studies [17,23]. These risk factors contribute to the dysfunctional placental development that ultimately leads to the occurrence of IUGR by limiting the placental exchange capacity [24].

Recently, Miglioli et al. conducted a prospective cohort study that evaluated the hypothesis of altered fetal brain functional connectivity in fetuses with an increased risk of preterm birth [25]. The authors used functional magnetic resonance imaging for scanning the brains of 31 singleton fetuses between 28 and 34 weeks gestational age, with high or low risk of preterm birth, and the collected data were included in random forest algorithms that indicated an increased risk of preterm birth in case of fewer fetal brain functional connections.

The continuous improvement in the neonatal care of premature and growth-restricted newborns has led to the minimization of adverse neonatal outcomes associated with this disorder. These changes are reflected by our results, which indicated that premature newborns with IUGR had significantly higher gestational age at birth compared with premature newborns without IUGR. Moreover, we found no statistically significant differences between these groups regarding Apgar scores at 1 and 5 min, as well as the hospitalization duration. However, we must outline that the main neonatal outcomes, such as mean Apgar scores at birth, were low, while the acute respiratory distress rates were high for both groups, thus requiring the admission to the neonatal intensive care unit.

Previous literature has cited higher rates of adverse neonatal outcomes in growth-restricted newborns. A retrospective cohort study by Chu et al. included patients with or without IUGR who received retinopathy of prematurity screening in a level IV neonatal intensive care [26]. Their results indicated that IUGR infants were more likely to have a worse stage of retinopathy of prematurity and treatment-requiring retinopathy of prematurity compared to non-IUGR infants.

The intraventricular hemorrhage rates were higher in the IUGR group, even though the differences were not statistically significant. A recent study by Misan et al. assessed the endothelial damage in the thigh junctions as well as the brain-sparing effect in pregnancies complicated by IUGR [27]. The authors found out that the IUGR newborns with centralized circulation were about 20 times more likely to develop an intraventricular hemorrhage (IVH) than the IUGR infants without this change.

Moreover, a prospective cohort study evaluated the risk of cranial ultrasound abnormalities, such as periventricular leukomalacia, intraventricular hemorrhage, and basal ganglia lesions in growth-restricted newborns and controls [28]. The results from this study indicated that growth-restricted newborns had a higher incidence of cranial ultrasound abnormalities, as well as an increased risk of mortality due to these lesions.

We could not determine any statistically significant differences between groups regarding the rates of prematurity retinopathy, intraventricular hemorrhage, and periventricular leukomalacia between groups. These findings could be explained by the fact that the control group also comprised preterm newborns admitted to the neonatal intensive care unit, who had high rates of complications and required specialized care; thus, the differences were significantly reduced between groups.

We could not find any statistically significant differences between groups regarding their Amiel-Tison evaluation at discharge and the Bayley-III evaluation at 2 years follow-up. A prospective study examined the association between the Amiel-Tison neurological assessment in preterm infants and their psychosocial functioning during adolescence [29].

The authors showed that among the three groups classified based on neurological signs as normal, intermediate, or abnormal, parents of adolescents with normal Amiel-Tison neurological assessment reported the fewest executive function problems and behavioral symptoms [29]. Additionally, the adolescents themselves reported the fewest behavioral symptoms and the highest quality of life. The authors concluded that utilizing this type of examination could be beneficial in clinical settings for identifying children who are at risk for future psychosocial issues and for the prevention of these problems by the implementation of early interventional programs.

The psychometric properties of the Bayley-III were examined by Yu et al. for both term and preterm infants [30]. A total of 47 full-term infants and 167 preterm infants were systematically assessed using the Bayley Scales of Infant Development—2nd Edition (BSID-II)— and the Bayley-III at 6, 12, 18, and 24 months of age (adjusted for prematurity). In this study, the authors found out that term infants outperformed preterm infants on all of the Bayley-III scales, with statistically significant differences, and concluded that the Bayley-III is a dependable tool that enhances its previous version, particularly in the evaluation of early language skills.

In our study, the rates of severe cognitive, language, and motor delays were low, and our results are comparable to previously published data. Furthermore, Ballot et al. carried out a cohort follow-up study to assess the proficiency of a sample of typical inner-city children in South Africa by comparing their performance on the Bayley-III assessment with that of the Bayley normative population using a cut-off of either 70 or 85 to define handicap [31]. According to their findings, none of the children exhibited any signs of developmental delay when utilizing a threshold score of 70. These findings suggest that some cut-offs need adjustments for various populations. This aspect was confirmed in a recent cross-sectional pilot study that enrolled 270 infants between 18 and 42 months of age from Egypt who underwent assessment of cognitive, language, and motor skills using the Bayle-III scale [32]. The results were compared to the American norm scores. The study showed that the mean cognitive, language, and motor composite scores were significantly lower compared to the American mean scores.

Finally, we constructed a three-layered artificial neural network for the prediction of adverse neurodevelopmental outcomes in IUGR patients, considering the grading from the Bailey-III scale. Our results indicated that this type of neural network exhibited moderate predictive performance in predicting mild forms of cognitive, motor, and language delays. However, the accuracy of predicting moderate and severe neurodevelopmental outcomes varied between moderate and low. The best results were obtained for predicting moderate cognitive delays, with a sensitivity of 66.6%, specificity of 94%, false positive rate of 5%, and an accuracy of 85.7%. For predicting severe cognitive delays, the sensitivity was 50%, the specificity was 100%, the false positive rate was 0%, and the accuracy was 83.3%. The artificial neural network that was suggested exhibited the least accurate predicting ability when compared to cognitive and motor delays across all levels of language delay. Our results indicated that the need for mechanical ventilation, birthweight, and gestational age at birth were the most important features with an impact on the prediction of adverse neurological outcomes.

As far as we know, this is the first study that evaluated the predictive performance of a three-layered artificial neural network for the prediction of adverse neurological outcomes in preterm patients with IUGR; thus, comparable results are lacking in the literature. Our findings could be explained by the fact that severe neurodevelopmental delays are rarer and more unpredictable due to various factors that influence their occurrence in the postnatal period. Moreover, a certain degree of neurodevelopmental delay in preterm infants is expected to occur, but its evolution depends on various factors such as the degree of involvement from parents, environmental factors, access to specialized healthcare, etc.

Language delay was poorly predicted by our artificial neural network, and this might be due to the lack of inclusion of factors that intervene during the follow-up process, such as the parents’/caregivers’ involvement, the presence of siblings in the newborn’s place of residence, and the socio-economic status.

This study is subject to certain limitations, namely a small cohort of patients, a limited number of included parameters, and the lack of evaluation of the possible impact of antenatal corticosteroid administration on neurodevelopmental outcomes. Conversely, this study assessed how accurately a three-layered artificial neural network can predict neurodevelopmental outcomes of growth-restricted infants that were admitted to a neonatal intensive care unit in Romania. Additional research, with a greater number of patients, could ascertain the cost-effectiveness of this particular neural network for predicting neurodevelopmental outcomes in various clinical scenarios. Also, we plan to test the predictive performance of various types of artificial neural networks and machine learning-based algorithms that will include a higher number of features. 

## 5. Conclusions

Intrauterine growth restriction is an important cause of iatrogenic prematurity, and during the postnatal period, a variety of complications can occur.

The Bailey-III scale for the evaluation of neurodevelopmental delays in infants is a recognized tool that allows proper stratification of patients.

There is a constant need to improve the prediction of adverse neurodevelopmental outcomes in growth-restricted neonates who were born prematurely.

This study indicated that the predictive performance of a three-layered artificial neural network for the prediction of mild adverse neurodevelopmental outcomes in growth-restricted infants was moderate, while for severe neurodevelopmental outcomes it was low.

Further studies that will use various types of artificial intelligence-based techniques should include a large number of parameters, including factors that can influence neurodevelopmental outcomes during the postnatal follow-up.

## Figures and Tables

**Table 1:** Clinical characteristics of the evaluated mothers.

Clinical Characteristics	IUGR Group (n = 27 Patients)	Control Group (n = 179 Patients)	*p* Value
Age, years (mean ± SD)	24.15 ± 4.36	26.16 ± 3.28	0.34
BMI, kg/m^2^, (mean and standard deviation)	23.36 ± 2.61	23.48 ± 3.18	0.76
Level of education (n/%)	Primary school (≤4 years of study)-2 (7.4%) Pre-high school (5–8 years of study)-7 (25.93%) High-school (9–12 years of study)-12 (44.44%) ≥Bachelor degree-6 (22.22%)	Primary school (≤4 years of study)-20 (11.7%) Pre-high school (5–8 years of study)-62 (34.64%) High-school (9–12 years of study)-63 (35.20%) ≥Bachelor degree-34 (18.99%)	0.67
Smoking habit (n/%)	Yes =11 (40.7%)	Yes = 16 (8.93%)	<0.001
Vaginal infections (n/%)	Yes = 3 (11.11%)	Yes = 41 (22.9%)	0.16
Chorioamnionitis (n/%)	Yes = 1 (3.7%)	Yes = 9 (5.02%)	0.76
Prolonged rupture of membranes (n/%)	Yes = 1 (3.7%)	Yes = 10 (5.58%)	0.68
Diabetes (n/%)	Yes = 2 (7.4%)	Yes = 5 (2.79%)	0.21
Preeclampsia (n/%)	Yes = 15 (55.55%)	Yes = 14 (7.8%)	< 0.001
Abruptio placentae (n/%)	Yes = 2 (7.4%)	Yes = 4 (2.23%)	0.13
HELLP (Hemolysis, Elevated Liver enzymes, and Low Platelets) syndrome (n/%)	Yes = 1 (3.7%)	Yes = 3 (1.67%)	0.47
Maternal thrombophilia (n/%)	Yes = 1 (3.7%)	Yes = 5 (2.79%)	0.79
History of adverse pregnancy outcomes (n/%)	Yes = 5 (18.5%)	Yes = 10 (5.58%)	0.01

Legend: IUGR—intrauterine growth restriction, SD—standard deviation, n—number of patients, and BMI—body mass index.

**Table 2:** Comparison of neonatal outcomes between groups.

Neonatal Outcome	IUGR Group (n = 27 Patients)	Control Group (n = 179 Patients)	*p* Value
Gestational age at birth, weeks (median and IQR)	31 (30–32)	30 (28–32)	0.06
Birthweight, g (median and IQR)	1300 (1050–1400)	1400 (1020–1750)	0.04
Apgar score at 1 min (median and IQR)	5 (4–7)	5.5 (4–7)	0.92
Apgar score at 5 min (median and IQR)	7 (5–7)	7 (5–7)	0.95
ARDS (n/%)	Yes = 24 (88.8%)	Yes = 170 (94.97%)	0.20
Need for mechanical ventilation (n/%)	Yes = 16 (59.25%)	Yes = 53 (29.6%)	0.005
ROP (n/%)	Stage I-2 (7.41%) Stage II-1 (3.7%) Stage III-0 (0%)	Stage I-17 (9.5%) Stage II-17 (9.5%) Stage III-1 (0.55%)	0.35
IVF (n/%)	Grade I-5 (18.52%) Grade II-4 (14.81%) Grade III-0 (0%) Grade IV-1 (3.7%)	Grade I-29 (16.20%) Grade II-17 (9.5%) Grade III-4 (2.23%) Grade IV-0 (0%)	0.21
PVL (n/%)	Grade I-2 (7.41%) Grade II-1 (3.7%) Grade III-1 (3.7%) Grade IV-1 (3.7%)	Grade I-6 (16.20%) Grade II-0 (0%) Grade III-1 (0.55%) Grade IV-0 (0%)	0.06
Duration of hospitalization, days (mean ± SD)	46.25 ± 20.30	49.77 ± 29.30	0.27

Legend: IUGR—intrauterine growth restriction, SD—standard deviation, IQR—interquartile range, n—number of patients, ROP—retinopathy of prematurity, IVH—intraventricular hemorrhage, PVL—periventricular leukomalacia, and ARDS—acute respiratory distress syndrome.

**Table 3:** Comparison of neurological and neurodevelopmental outcomes between groups.

Neonatal Outcome	IUGR Group (n = 27 Patients)	Control Group (n = 179 Patients)	*p* Value
Amiel Tison scale at discharge (n/%)	Mild-6 (22.22%) Moderate-16 (59.25%) Severe-5 (18.51%)	Mild-34 (18.99%) Moderate-111 (14.81%) Severe-34 (62.01%)	0.92
Bailey-III scale evaluation at 24 months considering CC score	Mild-4 (14.81%) Moderate-2 (3.7%) Severe-1 (3.7%)	Mild-20 (11.17%) Moderate-14 (7.82%) Severe-1 (0.55%)	0.42
Bailey-III scale evaluation at 24 months considering LC score	Mild-1 (3.7%) Moderate-4 (14.81%) Severe-3 (11.11%)	Mild-5 (2.79%) Moderate-54 (30.16%) Severe-26 (14.52%)	0.30
Bailey-III scale evaluation at 24 months considering MC score	Mild-7 (25.92%) Moderate-3 (11.11%) Severe-1 (3.7%)	Mild-32 (17.87%) Moderate-13 (7.26%) Severe-2 (1.11%)	0.38
Bailey-III scale evaluation at 24 months considering mixed delays	Mild-1 (3.7%) Moderate-1 (3.7%) Severe-0 (0%)	Mild-7 (3.91%) Moderate-5 (2.79%) Severe-0 (0%)	0.47

Legend: IUGR—intrauterine growth restriction, SD—standard deviation, n—number of patients, CC—Cognitive Composite, LC—Language Composite, and MC—Motor Composite.

**Table 4:** The predictive performance of a three-layered artificial neural network for the prediction of adverse neurodevelopmental outcomes at a 2-year follow-up.

Neurodevelopmental Outcome	Grade	Se (%)	Sp (%)	FPR (%)	Matthews Coefficient	Accuracy (%)	Precision	F1 Score	Mean_Se_	Mean_Sp_	Mean_Acc_
Cognitive delay	Mild	75	66.6	33.3	0.41	71.4	0.75	0.75	79.89	77.18	80.54
	Moderate	66.6	94	5	0.73	85.7	0.85	0.73	84.55	81.42	85.29
	Severe	50	100	0	0.63	83.3	1	0.66	62.76	80.51	73.30
Motor delay	Mild	75	90	10	0.66	83.3	0.85	0.8	85.03	82.14	85.72
	Moderate	40	88.8	11	0.33	71.4	0.66	0.5	68.06	66.36	68.46
	Severe	20	87.5	12.5	0.10	61.5	0.5	0.28	57.73	55.94	58.16
Language delay	Mild	62.3	87.5	12.5	0.37	80	0.5	0.5	78.50	76.07	79.08
	Moderate	60	85.7	14.2	0.47	75	0.75	0.66	74.80	73.06	75.95
	Severe	40	83.3	16.6	0.26	63.6	0.66	0.5	63.85	61.87	64.32

Legend: IUGR—intrauterine growth restriction, Se—sensibility, Sp—specificity, FPR- False positive rate.

## Data Availability

The data presented in this study are available on request from the corresponding author. The data are not publicly available due to local policies.
